# Maternal fibre and gluten intake during pregnancy and risk of childhood celiac disease: the MoBa study

**DOI:** 10.1038/s41598-020-73244-4

**Published:** 2020-10-02

**Authors:** Nicolai A. Lund-Blix, German Tapia, Karl Mårild, Anne Lise Brantsæter, Merete Eggesbø, Siddhartha Mandal, Lars C. Stene, Ketil Størdal

**Affiliations:** 1 Department of Chronic Diseases and Ageing, Division for Mental and Physical Health, Norwegian Institute of Public Health, Oslo, Norway; 2 Department of Pediatric and Adolescent Medicine, Oslo University Hospital, Oslo, Norway; 3 Department of Pediatrics, The Sahlgrenska Academy at University of Gothenburg and Queen Silvia Children’s Hospital, Gothenburg, Sweden; 4 Department of Environmental Exposure and Epidemiology, Norwegian Institute of Public Health, Oslo, Norway; 5 Center for Chronic Disease Control, New Delhi, India; 6 Department of Pediatrics, Østfold Hospital Trust, Grålum, Norway; 7 Department of Pediatric Research, Division of Paediatric and Adolescent Medicine, Oslo University Hospital, Oslo, Norway

**Keywords:** Coeliac disease, Paediatric research

## Abstract

Maternal diet can influence the developing immune system of the offspring. We hypothesized that maternal fibre and gluten intake during pregnancy were associated with the risk of celiac disease in the child. In the Norwegian Mother, Father and Child Cohort Study (MoBa, n = 85,898) higher maternal fibre intake (median 29.5 g/day) was associated with a lower risk of celiac disease in the offspring (adjusted relative risk 0.90, 95% CI 0.83 to 0.98 per 10 g/d increase). Gluten intake during pregnancy (median 13.0 g/d) was associated with a higher risk of childhood CD (adjusted relative risk = 1.21, 95% CI 1.02 to 1.43 per 10 g/d increase). These results were largely unaffected by adjustment for the child’s gluten intake at 18 months. In an independent study of 149 mother/child dyads, maternal fibre intake did not predict concentrations of total or sub-types of short-chain fatty acids in repeated infant stool samples, or fecal microbiome diversity in the mother or child. Our results suggest that high fibre and low gluten intake during pregnancy could be protective factors for celiac disease, although the mechanism is unknown.

## Introduction

Celiac disease (CD) is a prevalent immune-mediated disease with onset commonly during childhood. Genetic factors have been shown to have a strong influence on the individual risk, but they cannot explain the rapid increase in prevalence confirmed by serological screening studies performed over time^1,2^. Environmental factors underlying this increase are largely unknown. Changes in dietary habits over time, such as intake of gluten and dietary fibre are among the potential environmental risk factors.

Serial screening of children at risk of CD has demonstrated that antibodies usually develop during the first years of life, often several years before clinical symptoms arise^3^. This motivates the study of environmental factors that operate early in life as potential triggers for the disease.

We and other researchers have recently shown in cohort studies that a higher amount of gluten during early childhood may increase the risk for CD^4–6^. In the PreventCD trial, gluten intake overall did not predict later CD but was positively associated with CD risk in a subgroup with the HLA-DQ2.5/DQ7 haplotype^7^. A low intake of refined cereals and sweet beverages at age 1 year has been associated with a reduced risk of celiac disease autoimmunity by age six years^8^. This may suggest that not only the individual’s gluten intake but also other dietary factors may modify the risk of disease. Gluten and dietary fibre intake are potential modulators of the individuals’ gut microbiota^9,10^. Metabolites derived from the intestinal microbes, especially short-chain fatty acids (SCFA), are associated with dietary fibre^10,11^. Whether the microbiome and it’s metabolites affects the risk of CD is still unclear^12^.

Maternal diet can influence the developing immune system of the offspring. In animal studies, high-fibre diet has been shown to regulate the maturation of immune cells ^13^, protect against airway inflammation ^14^ and autoimmune diabetes^13^. A recent cohort study showed a positive association between maternal gluten intake and offspring type 1 diabetes risk^15^ which could however not be replicated in a study from our group^16^. In a multicenter study, gluten intake the last month of pregnancy collected by recall 3–4.5 months after delivery was not associated with the risk of CD in the offspring^17^. To the best of our knowledge maternal fibre intake has not been studied in the context of offspring CD.

We aimed to study whether maternal gluten and fibre intake during pregnancy were associated with the risk of CD in children. As a secondary aim, we explore whether maternal fibre intake influenced the levels of short-chain fatty acids and microbiome diversity in the offspring’s stool samples.

## Results

**Figure 1:**
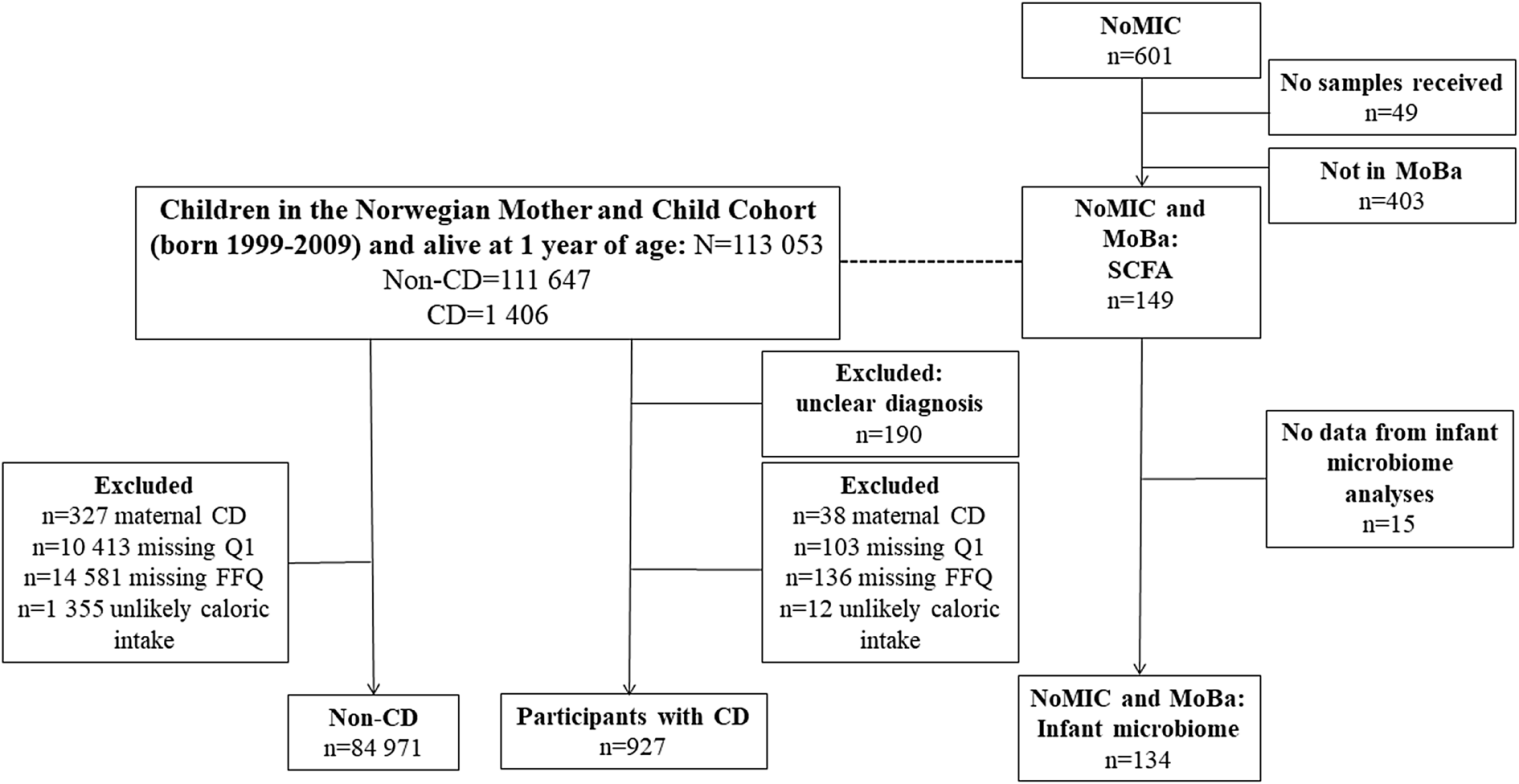
Flow chart for participation in MoBa and NoMic, two partly overlapping cohorts. MoBa: Norwegian Mother, Father and Child Cohort study; NoMIC: Norwegian Microbiota Study; CD: Celiac disease; Q1: Questionnaire 1 (at inclusion, 17 weeks of gestation); FFQ: Food Frequency Questionnaire; SCFA: Short Chain Fatty Acids.effecef.

Among 85,898 children in the Norwegian Mother, Father and Child Cohort study (MoBa) with complete exposure data from pregnancy, we identified 927 (1.1%, 62% female) who were diagnosed with celiac disease during a mean observational time of 11.0 years (range 7.5–16.5, flow chart in Fig. 1).

### Gluten and fibre intake in pregnancy

The mean daily intake of gluten among mothers during the first half of pregnancy was 13.5 g (SD 5.0), corresponding to approximately six slices of bread (40 g). The mean total fibre intake was 31.0 g/day (SD 10.5) (details in Supplemental Table 1 and Table 1, respectively). The national recommendations for minimum fibre intake of 25 g/day^18^ was reached by 70% of participants. Cereal fibre constituted 56% (17.4 g/day), vegetables/legumes/potatoes 14% (4.3 g/day) and fruits/berries 21% (6.6 g/day) of the total fibre intake. Total fibre and gluten intake were positively correlated (Spearman’s rho 0.70, *p* < 0.001).

**Table 1:** Baseline characteristics in MoBa participants (n = 85,898) by fibre intake in centile groups.

	Estimated daily fibre intake from conception to week 22 of pregnancy						
	< 10th centile n = 8,581	10–20 centile n = 8,600	20–50 centile n = 25,754	50–80 centile n = 25,810	80–90 centile n = 8,581	> 90th centile n = 8,572	*p *Vvalue ^5^
**Maternal age (%)**							< 0.001
< 25	1,524 (17)	1,025 (11)	2,440 (27)	2,219 (25)	789 (9)	925 (10)	
25–34	5,972 (10)	6,235 (10)	18,804 (30)	18,685 (30)	6,109 (10)	5,901 (10)	
≥ 35	1,085 (7)	1,340 (9)	4,510 (30)	4,906 (32)	1,683 (11)	1,746 (11)	
**Maternal education, (%)**^**1**^							< 0.001
≤ 12 years	4,093 (13)	3,355 (11)	8,789 (29)	8,156 (27)	2,939 (10)	3,343 (11)	
12–15 years	3,065 (9)	3,444 (10)	10,877 (31)	10,947 (31)	3,492 (10)	3,236 (9)	
≥ 16 years	1,380 (7)	1,761 (9)	5,982 (30)	6,603 (33)	2,105 (11)	1,936 (10)	
**Maternal smoking during pregnancy, (%)**^**2**^							< 0.001
No	7,461 (10)	7,741 (10)	23,510 (30)	23,779 (30)	7,869 (10)	7,735 (10)	
Occasionally	174 (12)	151 (10)	412 (28)	424 (29)	135 (9)	154 (11)	
Yes	897 (15)	667 (11)	1,707 (29)	1,477 (25)	523 (9)	623 (11)	
**Parental celiac disease, (%)**^**3**^							0.92
	91 (11)	80 (9)	260 (30)	258 (30)	90 (10)	80 (9)	
Child							
**Female sex (%)**							0.87
	4,236 (10)	4,165 (10)	12,587 (30)	12,607 (30)	4,164 (10)	4,187 (10)	
**Age end 2016, mean (SD)**							< 0.001
	11.1 (1.9)	11.1 (1.9)	11.1 (1.9)	11.0 (1.9)	11.0 (1.9)	10.9 (1.9)	
**Tertile for gluten intake at 18 months**^**4**^							< 0.001
1st (low)	2,809 (13)	2,590 (12)	6,784 (32)	5,808 (27)	1,820 (8)	1,720 (8)	
2nd	1,994 (9)	2,252 (10)	6,819 (31)	6,655 (31)	2,114 (10)	1,879 (9)	
3rd (high)	1,464 (7)	1,694 (8)	6,239 (29)	7,139 (33)	2,513 (12)	2,628 (12)	

SD: Standard deviation.

Fibre intake median 29.5, interquartile range 23.7–36.6.

Fibre intake < 10 centile: < 19.0 g/day.

Fibre intake 10–20 centile: 19.0–22.4 g/day.

Fibre intake 20–50 centile: 22.4–29.5 g/day.

Fibre intake 50–80 centile: 29.5–38.7 g/day.

Fibre intake 80–90 centile: 38.7–44.8 g/day.

Fibre intake > 90 centile: > 44.8 g/day.

^**1**^Missing variable for education: n = 395.

^**2**^Missing variable for smoking: n = 459.

^3^Diagnosis of incident celiac disease after pregnancy in mother or father.

^4^Missing variable for gluten intake at 18 months: n = 20,977.

^5^Chi-square test for categorical variables, t-test for continuous variables.

Mothers with higher intake of fibre tended to be older, non-smokers and with higher education (*p* < 0.001, Table 1). However, the differences in these background characteristics across categories of fibre and gluten intake were small (Table 1 for fibre and Supplemental Table 1 for gluten intake).

### Maternal gluten intake was associated with offspring CD

**Figure 2:**
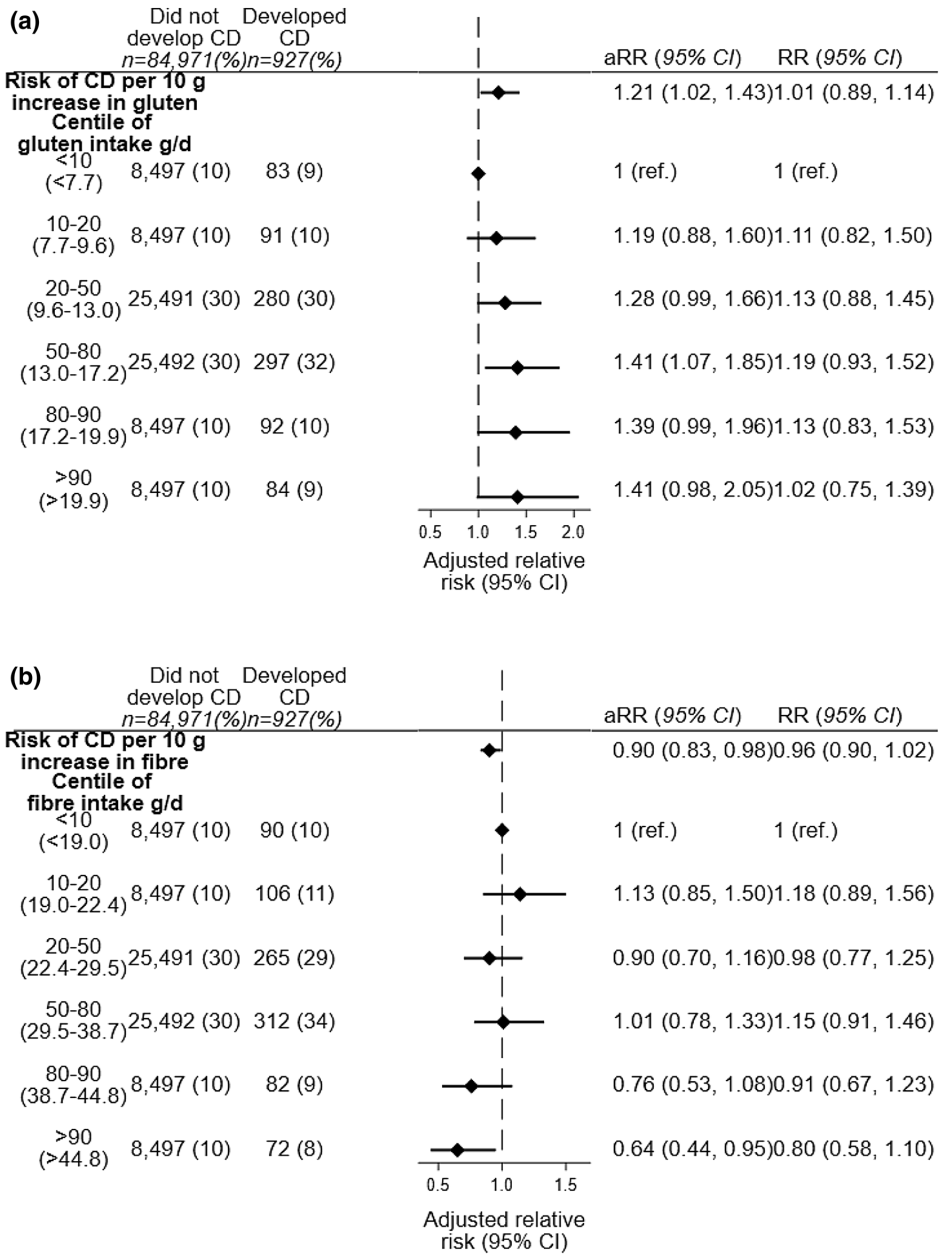
(**a**) Forest plot of risk for CD by gluten intake during pregnancy. (**b**) Forest plot of risk for CD by fibre intake during pregnancy. CD: Celiac disease; RR: Relative risk; CI: Confidence Interval; g/d: grams/day. Adjusted for child sex and age, parental celiac disease (CD if diagnosed after pregnancy) and reciprocally for maternal gluten and fibre intake.

Maternal gluten intake was associated with the risk of celiac disease in the offspring in adjusted continuous exposure analyses (adjusted relative risk (aRR) 1.21, 95%CI 1.02–1.43 per 10 g/day increase, *p* = 0.031, Fig. 2a). In categorical analyses the relative risk (RR) for CD in the highest compared to the lowest category of gluten intake was 1.41 (95% CI 0.98–2.05) and across centiles the P_trend_ was 0.028. Among the covariates we adjusted for, adjustment for maternal fibre intake level was the single covariate that resulted in the largest change in relative risk estimate (for the maternal gluten – child CD association) when comparing unadjusted (RR = 1.01) and adjusted (RR = 1.15) point estimates.

In additional analyses including the child’s gluten intake (available for 65,188 children)^5^, both maternal and child gluten intake were positively associated with the risk of offspring CD (aRR 1.26, 95%CI 1.03–1.54 per 10 g/day increase in maternal gluten intake, Supplemental Table 2). The RR did not change substantially after adjustment for maternal socioeconomic and lifestyle factors (Supplemental Table 2).

### Higher maternal fibre intake was associated with lower risk for offspring CD

Increasing maternal fibre intake was associated with a lower risk of celiac disease in adjusted analyses (aRR 0.90, 95% CI 0.83–0.98 per 10 g/d fibre intake, Fig. 2b). The relative risk of CD in children of mothers in the highest centile of fibre intake compared with the lowest centile was 0.64 (95% CI 0.44–0.95), and with a significant inverse association across centiles of fibre intake (P_trend_ 0.020).

In additional analyses with adjustment for the child’s gluten intake, the measures of association between maternal fibre intake and offspring CD were slightly strengthened (Table 2). Additional adjustment of the observed inverse association for fibre intake and risk of CD for maternal lifestyle characteristics (smoking, pre-pregnant BMI) or socioeconomic variables (education, maternal age at birth) changed the risk estimates minimally (Table 2).

**Table 2:** Additional adjustment models for maternal fibre intake and risk of CD in the offspring (n = 85,891). Relative risk per 10 g/day increase in maternal fibre intake.

Model	Adjusted relative risk (95% CI)	*p *Value
Main*	0.90 (0.83–0.98)	0.020
**Additional adjustment for:**		
Maternal education^1^	0.90 (0.82–0.98)	0.020
Maternal age	0.91 (0.83–0.99)	0.025
Maternal smoking^2^	0.90 (0.82–0.98)	0.017
Maternal pre-pregnant BMI^3^	0.91 (0.83–0.99)	0.038
Child gluten intake^4^	0.88 (0.80–0.98)	0.020

*Adjusted for chld age, sex, parental CD and gluten intake during pregnancy.

^1^Missing n = 395.

^2^Missing n = 459.

^3^Missing n = 2,187.

^4^Missing n = 20,977.

When taking into account the dietary sources of fibre, fibre from fruits and berries was associated with a lower risk of CD (aRR 0.984 per g/day increase, 95% CI 0.971–0.999, *p* = 0.025) whereas fibre from cereals or vegetables were not (Table 3).

**Table 3:** Risk of CD in the offspring by source of maternal fibre intake (n = 85,891). Relative risk per g/day increase in fibre intake.

Fibre source, per g increase of daily intake	Unadjusted relative risk (95% CI)	Adjusted relative risk* (95% CI)	*p *Value
Total*	0.996 (0.989–1.002)	0.990 (0.981–0.998)	0.020
Cereals^1^	0.999 (0.990–1.007)	0.988 (0.969–1.006)	0.188
Vegetables, legumes^2^	0.985 (0.958–1.012)	0.984 (0.954–1.014)	0.293
Fruits^3^	0.988 (0.976–1.000)	0.984 (0.970–0.998)	0.023

*Adjusted for child age, sex, parental CD and gluten intake during pregnancy.

^1^Contributed to a mean of 17.4 g/day (56% of total fibre).

^2^Contributed to a mean of 4.3 g/day (14% of total fibre).

^3^Contributed to a mean of 6.6 g/day (21% of total fibre).

### Maternal fibre intake was not associated with levels of short-chain fatty acids in infant stools

The participants of both the MoBa and the NoMIC study (n = 149) did not differ in baseline characteristics from those who participated in NoMIC only (Supplemental Table 3). In repeated stool samples from infants (40 to 367 samples at six different time-points, Supplemental Fig. 1) from age 4 days to 2 years, the predominant types of the eight SCFAs increased by age. The total concentration of short-chain fatty acids (SCFA) was not associated with the mothers fibre intake during pregnancy (β -0.037, 95% CI -0.22 to 0.18, *p* = 0.62).

#### Maternal fibre intake was not associated with microbiome diversity in maternal or infant stools

Fibre intake did not predict the microbiome α-diversity in maternal stool samples (Supplemental Table 4). For infant stool samples, we similarly did not find any association between high versus low maternal fibre intake and α-diversity, though a higher diversity was observed at age two years in offspring of mothers with high fibre intake (Supplemental Table 4).

## Discussion

In this large-scale prospective cohort study, children of mothers with high fibre intake during early pregnancy had lower risk of celiac disease. Maternal gluten intake was positively associated with the risk of offspring CD. To the best of our knowledge, this is the first study of maternal fibre intake during pregnancy and CD.

The main strengths of this study are the large sample size and population base, and the prospective design avoiding recall bias. A validated food frequency questionnaire was used, though validation against weighed food diaries has not been performed specifically for gluten and fibre^19^. The validation study demonstrated that relative to a dietary reference method, motion sensor and several biological markers, the food frequency questionnaire produced a realistic estimate of the habitual intake and is a valid tool for ranking pregnant women according to high and low intakes of energy, nutrients, and food^19,20^. However, the food frequency method is rather imprecise and there will always be uncertainty in the intake estimates. Thus, the estimated intake of gluten and fibre from our study should thus not be translated directly into recommendations. Furthermore, imprecision in the intake estimates may result in attenuation of associations with the outcome.

With information on a number of covariates, we explored and adjusted for several potential confounders including infant feeding practices. As in any observational study, the possibility of residual confounding can, however, not be completely ruled out. High fibre intake may be seen as a marker of a healthy lifestyle, and thus confounded by other healthy choices. Even though the reported intake of dietary fibre varied to some extent by socioeconomic factors, adjustment for lifestyle and socioeconomic factors in our study had only a small impact on the estimates.

The self-selection of participants in such a study could limit the external validity of our findings. MoBa participants tend to be older, better educated and with fewer smokers than the average population of women giving birth in Norway. However, assessing well established associations in MoBa compared to the population has demonstrated that associations were robust to a potential selection bias^21^.

We did not have access to antibody screening among the participants. Certainly, antibody screening of the cohort would have reclassified a proportion of the controls as cases, likely around 1% of the cohort if the prevalence of undiagnosed CD is comparable to other Nordic countries^22,23^. However, such potential misclassification would only bias our results toward no association. In conditions with undetected true cases, the risk of false negatives is a smaller threat against validity than false positives. Thus, the ESPGHAN criteria for CD were applied^24^, and we excluded cases with unclear diagnosis.

A previous prospective cohort study used recall of the last 1–2 months of pregnancy at infant age 3 months, and found no association between maternal gluten intake and offspring celiac disease^17^. Our study covered diet in the first half of pregnancy with data collection mid-pregnancy. Dietary patterns around one year of age was a significant predictor for CD in a recent study, with a lower risk in infants with a “prudent” diet consisting of less refined cereals and more fibre^8^.

### Potential mechanisms

In our study, we were able to explore differences in microbiome and fecal SCFA as potential mechanisms for the observed association in a sub-sample. In two randomized cross-over trials fibre intake was associated with higher levels of SCFA in stools and abundance of potentially beneficial phyla with anti-inflammatory features as bifidobacteria^25,26^. Whether this fibre-driven microbiome also is transferred from mother to infant is to our knowledge not studied. The child’s microbiome has been shown to be strongly associated to the maternal microbiome, likely by direct transfer during birth and not present in those delivered by Cesarean section^27–29^.

The fibre intake in infancy and onwards is likely correlated with maternal fibre intake. Speculatively, it seems plausible that fibre intake in the child in early life has a stronger association to CD risk than the maternal fibre intake. Unfortunately, our study contained data on only maternal fibre and the comparable study only on infant fibre^8^, and a direct comparison could not be done.

A causal relationship between the microbiota and CD has not been established, though the microbiome clearly has programming effects on the immune system through complex pathways^30^. Circumstantial evidence comes from studies indicating increased risk of CD after antibiotic use during infancy^31–33^, and from cross-sectional studies identifying specific bacteria in the duodenum at diagnosis^34,35^.

An alternative mechanism not directly dependent of the microbiome would be programming effects of metabolites on the maturing immune system. SCFA bind to proteins in the placenta, and experimental animal studies suggest that SCFA exert cross-placental anti-inflammatory effects^36–38^.

### Implications and future directions

If dietary factors beyond gluten shape the gut immunity and the risk of future CD, this could have implications for dietary advice during pregnancy and early life. Still these findings warrant corroboration, preferably in an intervention setting or else in cohort studies with dietary information in dyads of mothers and their offspring. In daily life, high fibre and low gluten intake may be challenging. However, our data suggest that high fruit fibre intake, and not fibre from cereals, was driving the negative association with CD. Given the modest association, other observational studies would be of interest before any interventional studies are considered.

In conclusion, pregnant women with a higher fibre intake had a lower risk of CD in their offspring. Higher maternal gluten intake was associated with CD. The maternal fibre intake was not a predictor of infant microbiome diversity and SCFA levels in stool samples.

## Material and methods

We used information from the population-based Norwegian Mother, Father and Child Cohort Study (MoBa)^39^. Participating mothers were recruited across Norway during mid-pregnancy from 1999–2008 after written informed consent, and their offspring were followed for celiac disease to 1st of January 2017. The establishment of MoBa and initial data collection was based on a license from the Norwegian Data protection agency and approval from The Regional Committees for Medical and Health Research Ethics. The MoBa cohort is based on regulations based on the Norwegian Health Registry Act. The current study is based on MoBa version VIII of the quality-assured data files released for research in February 2014.

Children whose mothers returned the food frequency questionnaire used to estimate the daily intake of gluten and fibre were eligible (questionnaires available at https://www.fhi.no/en/studies/moba/for-forskere-artikler/questionnaires-from-moba/). Overview of included participants is provided in Fig. 1, and characteristics of participants in Table 1. We excluded mothers with an unlikely energy intake (< 4500 or > 20 000 kJ/day) and mothers who had celiac disease before completion of the recruitment questionnaire due to the influence on the maternal gluten intake, and potentially the child’s gluten intake.

### Outcome: Celiac disease diagnosis in childhood

We used codes from International Classification of Diseases (ICD-10) from the Norwegian Patient Register (NPR) to identify children with celiac disease (K90.0). Virtually all specialist care for children is reimbursed based on diagnoses reported to the NPR since 2008, and a specialist is required to confirm the diagnosis. Follow-up by a pediatrician after diagnosis is recommended. To avoid false positives our case definition requires a minimum of two entries with K90.0 in NPR, because a working diagnosis may be given before complete diagnostic evaluation.

As an additional source of diagnosis, the questionnaire in MoBa at age seven and eight asks “does your child have celiac disease”. Celiac disease reported from parental questionnaires were classified as cases, irrespective of whether they were also identified in NPR or not. Those with a single entry only in NPR were excluded due to unclear status (Fig. 1). In a validation of CD identified in MoBa by 2014, > 92% of the children meeting this case definition were confirmed by their parents to have CD ^40^. The ESPGHAN criteria from 2012 were applied nation-wide for a diagnosis of CD^24^, and the large majority of cases CD had diagnosis confirmed by biopsy ^40^.

### Norwegian microbiota study—NoMIC

An independent study (NoMIC, https://www.fhi.no/en/studies/nomic) provided stool samples from the mother and from the infant. A sub-cohort of mothers who participated in the MoBa study as well as in the NoMIC study, with linked data between these cohorts, was used to study the association between maternal fibre intake (< / > the median), microbiome diversity of the mother and the infant, and stool metabolites in the infant. We studied fecal samples collected at 4 days postpartum from the mother and from the child at age 4, 10 and 30 days, and at ages 4, 12 and 24 months^41^.

### Stool samples and analyses

DNA was extracted from the fecal samples, and subjected to barcoded sequencing of the V4 region of the 16S rRNA gene using an Illumina HiSeq 2000 machine^42^.

Samples were available from 40–367 infants at the respective time points (Supplement Fig. 1) for chemical analyses of eight short chain fatty acids (SCFA). SCFAs were analyzed by gas chromatography in two different laboratories (details are provided in ^43^).

### Main exposures: fibre and gluten intake during pregnancy

Around week 22 of pregnancy, the participating mothers returned a validated food frequency questionnaire asking about their average intake (usual diet) of 255 foods and dishes from the time around conception^19^. Qualified dieticians in the research group used the FoodCalc nutritional analysis software and the Norwegian food composition table to estimate average daily intake of foods and nutrients^44,45^. We converted the protein content from wheat-, barley- and rye-containing flour or grains from each of the food variables into gluten content by using a conversion factor of 0.75 in accordance with most studies^46–48^, and summarized into total gluten intake in grams per day.

FoodCalc was also used to estimate the daily intake of dietary fibre. Fibres are carbohydrate polymers with a minimum count of three monomeric units which are not digested in the small intestine^49^. The total fibre content (g/day) was estimated and further subdivided by food source into the three main categories: fibre from cereals, from fruits/berries, and from vegetables/legumes/potatoes.

### Other variables

From the Medical Birth Registry we obtained perinatal variables categorized as shown in Table 1. The Medical Birth Registry (MBRN) is a national health registry containing information about all births in Norway. The MoBa recruitment questionnaire provided information regarding maternal education, smoking, pre-pregnant body mass index (BMI) and maternal CD diagnosis. As previously described, we obtained the child’s gluten intake from the questionnaire completed at 18 months^5^. Linked data from the NPR, in addition to CD in the child, provided information on medical codes indicative of CD in the mother or father.

### Adjusted analyses

We decided a priori to include variables that may be associated with the main exposures (maternal gluten and fibre intake) and outcome (CD). In the main model we adjusted for incident maternal celiac disease (diagnosed after recruitment) and child sex and age at the end of study (January 1, 2017).

In additional analyses we tested whether proxies for maternal socioeconomic status (maternal education and age at delivery) and healthy lifestyle choices (pre-pregnant BMI and smoking) confounded the associations, defined as associated with both the exposure and outcome and causing a change in the relative risk estimates of > 10%. (DAG, Supplement Fig. 2). Finally, in 64,921 children with data on gluten intake at age 18 months additional analyses were performed including this covariate into our adjusted model.

### Statistical methods

We used binary regression to estimate relative risks, and included the maternal CD and child age/sex as covariates in a priori multivariable models. We hypothesized a linear association, but also explored potential non-linear associations by studying maternal gluten and fibre intake in centile groups of intake level (< 10, 10–20, 20–50, 50–80, 80–90 and > 90 centile). We used clustered sandwich estimator for variance to account for potential clustering among siblings.

Association of fibre intake (dichotomized into high and low intake) with microbial alpha diversity (measured by Shannon’s diversity) was analyzed using linear regression models at each time point with fibre intake as the only explanatory variable (Supplemental Table 4). We used longitudinal mixed effects models to analyze the associations between fibre intake and concentration of each short chain fatty acid (SCFA). Each model included an indicator variable for high fibre intake, time (in days) as fixed effects, a random intercept for each individual and a random slope for time within each individual. Effect estimates and associated 95% confidence intervals reported as part of these analyses indicate the average change in the specific acid concentration for high fibre intake compared to low fibre intake.

## Supplementary information

Supplementary file1

## Data Availability

The consent given by the participants does not open for storage of data on an individual level in repositories or journals. Researchers who want access to data sets for replication should submit an application to datatilgang@fhi.no. Access to data sets requires approval from The Regional Committee for Medical and Health Research Ethics in Norway and a formal contract with MoBa.
