# Supplementary material for: Maternal fibre and gluten intake during pregnancy and risk of childhood celiac disease: the MoBa study
Source: Sci Rep. 2020 Oct 2;10:16439. doi: 10.1038/s41598-020-73244-4 (PMC7532434; doi:10.1038/s41598-020-73244-4)
Supplement: Supplementary file 1 — Supplementary file1 [file 41598_2020_73244_MOESM1_ESM.docx]

**Online supplement**

This appendix has been provided by the authors to give additional information about their work.

Supplement to: Nicolai A. Lund-Blix, German Tapia, Karl Mårild, Anne Lise Brantsæter, Merete Eggesbø, Siddhartha Mandal, Lars C. Stene, Ketil Størdal.

**Maternal fibre and gluten intake during pregnancy and risk of childhood celiac disease: The MoBa Study**

**Table of contents**

[Supplemental Table 1: Baseline characteristics in participants (n=85,898) by gluten intake in centile groups. 2](#_Toc43370629)

[Supplemental Table 2: Additional adjustment models for maternal gluten intake and risk of CD in the offspring (n=85,891). Relative risk per 10 g/day increase in maternal gluten intake. 4](#_Toc43370630)

[Supplemental Table 3: NoMiC baseline characteristics of participants* with maternal dietary and maternal microbiota data (dietary data from MoBa due to concomitant participation in MoBa). Preterm deliveries were oversampled during recruitment to the study. 5](#_Toc43370631)

[Supplemental Table 4: Microbiota alpha diversity in stool samples by high versus low maternal fibre intake during pregnancy. 6](#_Toc43370632)

[Supplemental Fig 1: Concentrations of short chain fatty acids (SCFA) mmol/kg in infant stool samples from postnatal day 4-730 by maternal fibre intake above or below the median during pregnancy. 7](#_Toc43370633)

[Supplemental Fig 2: Directed Acyclic Graph of the analysis of maternal diet and offspring CD. 8](#_Toc43370634)

# Supplemental Table 1: Baseline characteristics in participants (n=85,898) by gluten intake in centile groups.

|  | Estimated daily gluten intake from conception to week 22 of pregnancy | | | | | |  |
| --- | --- | --- | --- | --- | --- | --- | --- |
|  | <10th centile  n=8,580 | 10-20 centile  n=8,588 | 20-50 centile  n=25,771 | 50-80 centile  n=25,789 | 80-90 centile  n=8,589 | >90th centile  n=8,581 | p-value ^5^ |
| Maternal |  |  |  |  |  |  |  |
| Age (%) |  |  |  |  |  |  | <0.001 |
| <25 | 1,285 (14) | 930 (10) | 2,393 (27) | 2,249 (25) | 893 (10) | 1,172 (13) |  |
| 25-34 | 5,871 (10) | 6,100 (10) | 18,685 (30) | 18,741 (30) | 6,224 (10) | 6,085 (10) |  |
| ≥35 | 1,424  (9) | 1,558  (10) | 4,693 (31) | 4,799 (31) | 1,472 (10) | 1,324 (9) |  |
| Education, (%)^1^ | | |  |  |  |  | <0.001 |
| ≤12 years | 3,862 (13) | 3,294 (11) | 8,766 (29) | 8,222 (27) | 2,908 (9) | 3,623 (12) |  |
| 12-15 years | 2,965 (8) | 3,414 (10) | 10,802 (31) | 11,077 (32) | 3,569 (10) | 3,234 (9) |  |
| ≥16 years | 1,692 (9) | 1,850 (9) | 6,095 (31) | 6,383 (32) | 2,070 (10) | 1,677 (8) |  |
| Smoking during pregnancy, (%)^2^ | | |  |  |  |  | <0.001 |
| No | 7,616 (10) | 7,773 (10) | 23,653 (30) | 23,636 (30) | 7,828 (10) | 7,589 (10) |  |
| Occasionally | 159 (11) | 163 (11) | 425 (29) | 404 (28) | 154 (11) | 145 (10) |  |
| Yes | 756 (13) | 598 (10) | 1,560 (26) | 1,611 (27) | 570 (10) | 799 (14) |  |
| Parental celiac disease, (%)^3^ | | | | |  |  | 0.042 |
|  | 113  (13) | 85  (10) | 233  (27) | 260  (30) | 86  (10) | 82  (10) |  |
| Child |  |  |  |  |  |  |  |
| Female sex (%) | |  |  |  |  |  | 0.37 |
|  | 4,370 (10) | 4,408 (10) | 13,119 (30) | 13,162 (30) | 4,408 (10) | 4,485 (10) |  |
| Age end 2016, mean (SD) | | |  |  |  |  | <0.001 |
|  | 10.9 (1.9) | 11.0 (1.9) | 11.0 (1.9) | 11.0 (1.9) | 11.1 (1.9) | 11.2 (1.9) |  |
| Tertile for gluten intake at 18 mo^4^ | | |  |  |  |  | <0.001 |
| 1st | 2,681 (12) | 2,513 (12) | 6,691 (31) | 5,870 (27) | 1,884 (9) | 1,892 (9) |  |
| 2nd | 1,955 (9) | 2,144 (10) | 6,793 (31) | 6,708 (31) | 2,172 (10) | 1,941 (9) |  |
| 3rd | 1,514 (7) | 1,807 (8) | 6,311 (29) | 7,106 (33) | 2,455 (11) | 2,484 (11) |  |

Gluten intake median 13.0, interquartile range 10.2 to 16.3

Gluten <10 centile: <7.7 g/day

Gluten 10-20 centile: 7.7-9.6 g/day

Gluten 20-50 centile: 9.6-13.0 g/day

Gluten 50-80 centile: 13.0-17.2 g/day

Gluten 80-90 centile: 17.2-19.9 g/day

Gluten >90 centile: >19.9 g/day

**^1^** Missing variable for education: n=395

**^2^** Missing variable for smoking: n=459

^3^ Diagnosis of incident celiac disease after pregnancy in mother or father.

^4^ Missing variable for gluten intake at 18 months: n=20,977

^5^ Chi-square test for categorical variables, t-test for continuous variables.

# Supplemental Table 2: Additional adjustment models for maternal gluten intake and risk of CD in the offspring (n=85,891). Relative risk per 10 g/day increase in maternal gluten intake.

| Model | Adjusted relative risk (95% CI) | p-value |
| --- | --- | --- |
| Main* | 1.21 (1.02-1.42) | 0.025 |
| Additional adjustment for: |  |  |
| Maternal education^1^ | 1.21 (1.02-1.44) | 0.026 |
| Maternal age | 1.20 (1.01-1.42) | 0.037 |
| Maternal smoking^2^ | 1.22 (1.03-1.45) | 0.023 |
| Maternal pre-pregnant BMI^3^ | 1.19 (1.00-1.41) | 0.038 |
| Child gluten intake^4^ | 1.26 (1.03-1.54) | 0.022 |

*Adjusted for child age, sex, parental CD and fibre intake during pregnancy.

^1^ Missing n=395

^2^ Missing n=459

^3^ Missing n=2,187

^4^ Missing n=20,977. Adjusted RR per standard deviation increase in child gluten intake 1.13 (1.06-1.21).

# Supplemental Table 3: NoMiC baseline characteristics of participants* with maternal dietary and maternal microbiota data (dietary data from MoBa due to concomitant participation in MoBa). Preterm deliveries were oversampled during recruitment to the study.

| NoMIC participants with dietary and microbiota data | | | |
| --- | --- | --- | --- |
|  |  | |  |
| Age, n (%) | Yes  n=134 | | No  n=418 |
| <25 | 17 (13) | | 45 (11) |
| 25-34 | 92 (69) | | 310 (74) |
| ≥35 | 25 (19) | | 63 (15) |
| Education^†^, n (%) | | |  |
| ≤12 years | 31 (23) | | 144 (33) |
| 12-15 years | 60 (45) | | 164 (47) |
| ≥16 years | 43 (32) | | 69 (20) |
| Smoking in pregnancy^‡^, n (%) | | | |
| No | 125 (93) | | 333 (82) |
| Occasionally | 2 (2) | | 16 (4) |
| Yes | 7 (5) | | 59 (14) |
| Cesarean section, n (%) | | |  |
|  | 44 (33) | | 138 (33) |
| Gestational age, n (%) | |  |  |
| <37 weeks | | 40(30) | 125 (30) |

NoMIC: Norwegian Microbiota Study

*601 subjects recruited to NoMIC where 552 returned samples and metadata.

**^†^** Missing for n=71 in the group without dietary data.

**^‡^** Missing for n=10 in the group without dietary data.

# Supplemental Table 4: Microbiota alpha diversity in stool samples by high versus low maternal fibre intake during pregnancy.

| Source | Time (days) |  | Sample size | Estimate | Standard error | p-value |
| --- | --- | --- | --- | --- | --- | --- |
| Mother |  |  |  |  |  |  |
|  | Delivery |  | 183 | -0.03 | 0.04 | 0.48 |
| Infant |  |  |  |  |  |  |
|  | 4 |  | 127 | 0.00 | 0.09 | 0.96 |
|  | 10 |  | 130 | 0.02 | 0.08 | 0.78 |
|  | 30 |  | 130 | -0.04 | 0.09 | 0.63 |
|  | 120 |  | 124 | 0.15 | 0.08 | 0.07 |
|  | 365 |  | 95 | -0.05 | 0.11 | 0.67 |
|  | 730 |  | 47 | 0.36 | 0.14 | 0.01 |

# Supplemental Fig 1: Concentrations of short chain fatty acids (SCFA) mmol/kg in infant stool samples from postnatal day 4-730 by maternal fibre intake above or below the median during pregnancy.

#


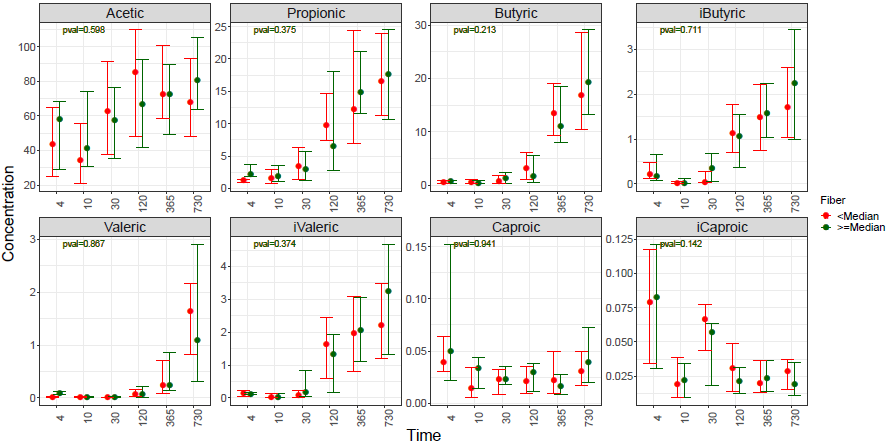


Number of samples analysed:

4 days: 40

10 days: 152

30 days: 123

120 days: 159

365 days: 367

730 days: 293

# Supplemental Fig 2: Directed Acyclic Graph of the analysis of maternal diet and offspring CD.


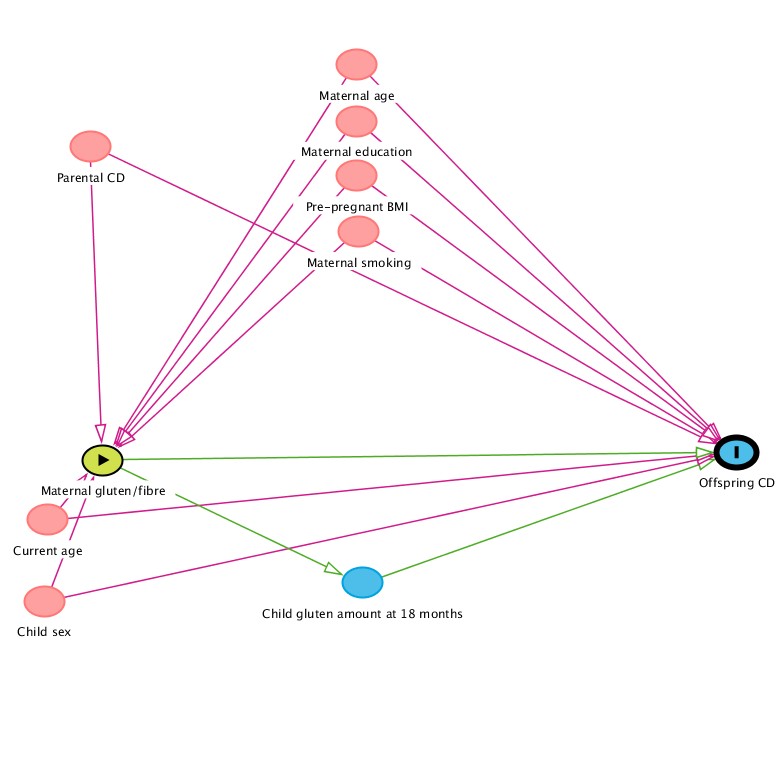


Green: Main exposure and causal path

Blue: Outcome or ancestor of outcome

Red: Adjustment variables. Primary model to the left, additional variables at the top of the diagram.
